# Spectral Spatial Variation

**DOI:** 10.1038/s41598-019-43971-4

**Published:** 2019-05-17

**Authors:** Martin Hohmann, Heinz Albrecht, Jonas Mudter, Konstantin Yu Nagulin, Florian Klämpfl, Michael Schmidt

**Affiliations:** 1 Friedrich-Alexander-Universität Erlangen-Nürnberg (FAU), Institute of Photonic Technologies (LPT), Konrad-Zuse-Straße 3/5, 91052 Erlangen, Germany; 2 Erlangen Graduate School in Advanced Optical Technologies (SAOT), Paul-Gordon-Straße 6, 91052 Erlangen, Germany; 3 Kliniken des Landkreises Neumarkt i.d.OPf., Department of Internal Medicine II, Nürnberger Str. 12, 92318 Neumarkt, Germany; 4 Sana Clinic Ostholstein, Department of Gastroenterology, Hospitalstraße 22, 23701 Eutin, Germany; 5 Kazan National Research Technical University named after AN Tupolev – KAI, Karl Marx Street 10, 420111 Kazan, Russia

**Keywords:** Oesophagogastroscopy, Biophotonics

## Abstract

Automatic carcinoma detection from hyper/multi spectral images is of essential importance due to the fact that these images cannot be presented directly to the clinician. However, standard approaches for carcinoma detection use hundreds or even thousands of features. This would cost a high amount of RAM (random access memory) for a pixel wise analysis and would slow down the classification or make it even impossible on standard PCs. To overcome this, strong features are required. We propose that the spectral-spatial-variation (SSV) is one of these strong features. SSV is the residuum of the three dimensional hyper spectral data cube minus its approximation with a fitting in a small volume of the 3D image. By using it, the classification results of carcinoma detection in the stomach with multi spectral imaging will be increase significantly compared to not using the SSV. In some cases, the AUC can be even as high as by the usage of 72 spatial features.

## Introduction

Gastric cancer is the second most frequent cause of cancer related death worldwide^1^. Despite many new technologies, high definition white light endoscopy (HD-WLE) is used for diagnosis in most cases. However, there is still a potential for improvement. One of the possible ways to further improve the accuracy of the carcinoma detection is virtual chromoendoscopy^2^. By means of spectral estimation, the virtual chromo endoscopy allows to generate images like chromo endoscopy. Newer meta analyses^3^ favour this method above all others. From the good results of virtual chromoendoscopy, Swager *et al*.^4^ concluded that spectroscopic quantitative measurements of tissue need further investigation in contrast to spectral estimations done by virtual chromoendoscopy. It is expected that spectroscopic quantitative measurements facilitates direct optical diagnosis of early neoplasia or risk stratification based on the presence of field carcinogenesis.

To take a step into the direction of direct spectroscopic quantitative measurements, we proposed multi spectral imaging (MSI) as a first step towards hyper-spectral video endoscopy (HSVE) in an earlier study^5^. However, there are still some problems for multi/hyper-spectral imaging. First, the exact margins of the carcinomas are not known for *in-vivo* situations and even medical experts cannot always correctly identify them^6^. However, RobustBoost (RB) seems to partly compensate this^5^. Second, inter-patient variations still strongly reduce the accuracy. The strong inter patient variations lead to a difficult transfer of the results.

Nevertheless, there are many approaches with all kinds of feature generation for the classification process to improve the classification results. One way of generalizing the classification process and thus increasing the classification results is the usage of spatial features. Many different approaches are used for decision support but only a low amount is used for carcinoma detection^7^. However, there is no analysis done for carcinomas of the upper gastro-intestinal tract (GI) in their review^7^. New research shows that it is possible to create a computer-aided method to identify images in the upper GI containing lesions with an accuracy of around 90% for early carcinomas in the upper GI^8^.

Despite these very good results generated by Liu *et al*.^8^, they can only identify that there is a lesion in an image but they cannot figure out where it is. Moreover, they need 1500 up to 10000 features for each image to do the analysis. Doing a similar analysis for multi/hyper-spectral images would generate more features as spatial features might change for different spectral bands. Furthermore, the ultimate goal is that the algorithm tells the clinician also where they should look for the carcinoma in an image. For this goal, a pixel by pixel analysis has to be done. A single endoscopic image consists of approximately 90,000 to 1,000,000 pixels. Having this amount of features for every pixel is impossible to handle in terms of memory and calculation time. Thus, it should also be noted that goal of the accuracy for the pixel by pixel analysis is lower due to the fact that so many feature dimensions are not possible.

Thus, a new set of strong features is required which contains relevant information in a low amount of features. For this, we propose combined spatial-spectral features generated from multi/hyper-spectral images. Spatial-spectral feature selection is already used in the analysis of remote sensing images^9^. However, they are just added to the selection of features where spectral features are one kind of features out of many. Another possibility is to use an adaptive neighbourhood system as Fauvel *et al*.^10^ do. However, the features do not summarize the spectral and spatial information of the spectral and spatial surrounding.

 Zhang *et al*.^11^ proposed the usage of the spatial-spectral surrounding. They suggested to use the tensor of the surrounding data points as input parameters. While this idea is good, for medical multi/hyper-spectral imaging it might lead to a too high dimensionality of the features space. Thus, this study presents a new spatial-spectral feature and its derivation which might be suited very well for medical multi/hyper-spectral imaging: the spatial-spectral variation (SSV).

## Material and Methods

### Patients

In this study, the same data is used as in our older study^5^. These are 14 patients with histopathologically confirmed adenoma carcinoma in the stomach. Eight out of these had undergone pretreatment. The youngest patient has an age of 50 years and the oldest has an age of 85 years. From these patients, eleven are male and three are female.

During the endoscopic investigation, the patients are kept under analgosedation to minimize anxiety and discomfort. The hyper spectral imaging is performed before the standard white light endoscopy procedure. Several images are taken from each patient with different angles and distances from the tip of the endoscope and the surface of the stomach to increase the amount of training data. From each patient, four to six biopsies are taken from each lesion.

#### Patient Consent

The patients received complete information about this study. Afterwards, the patients attested to informed consent for study participation. The research is carried out in accordance with the Declaration of Helsinki. The study has been approved by the Institutional Review Board (IRB) of the Friedrich-Alexander-Universität Erlangen-Nürnberg, Germany.

### Set-up

Due to the fact that the same data is used as in out old study^5^, the set-up is the same. The multi-spectral endoscopy set-up is a standard endoscopy system, consisting of an Olympus endoscope GIF 100 (Olympus Corporation, Tokyo, Japan), an Olympus video processor CV-140 (Olympus Corporation, Tokyo, Japan), a modified light source Olympus CLV-U40 (Olympus Corporation, Tokyo, Japan) and an external light source (Lumencor spectra 7-LCR-XA, Beaverton, OR, USA). The control of the external light source is done by a Matlab (The MathWorks, Inc., Narick, MA, USA) program.

The multi spectral image consists of six wavelength bands ranging from 400 to 650 nm. The multi spectral system is implemented as a spectral scanning system. The resolution is approximately 350 × 370 pixels. The field of view is 120 degrees. A sharp image can be generated with a distance of more than 3 mm between the tip of the endoscope and the surface. The depth of focus is 3–100 mm. In the images used for this study, the distance is most of the time between 5 mm and 50 mm. Hence, one pixel images an area with the size of 0.05 mm to 0.5 mm.

### Pre-processing

The pre-processing is mainly done the same way as in our previous study^5^ with three changes. The pre-processing still consists of a Gaussian filter for noise reduction and a principal component analysis (PCA) for feature reduction and further noise reduction. The new pre-processing steps are the following: First, the present barrel distortion of the endoscope^5^ is corrected to allow the spatial features and the spectral-spatial- variation to derive the same information over the whole image. Second, the noise filtering is changed to additionally use the minimum noise fraction (MNF) which is described in section 2.4. A Fourier filtering is introduced to remove the line artefacts caused by the endoscope.

In summary, there are six steps in the order shown in Table 1. Pre-processing steps done with the data. First, the Fourier-filtering is done. Afterwards the barrel distortion is corrected. Before the noise removal with MNF the images for every wavelength are normalized to better find the noise. After the noise removal, the data set is denormalized again. As last step, a Gaussian filter is applied to further reduce the noise. The Fourier filtering is done first, as the correction of the Barrel correction would alter the spatial frequencies of the line pattern artefacts. For the Fourier filtering lines with a spatial frequency of 0.5, 0.25 and 0.125 per pixel parallel to the x-axis are filtered out.

**Table 1:** Pre-processing steps done with the data.

Step	Pre-processing
1	Fourier filtering
2	Correction of the barrel distortion
3	Normalization
4	Noise filtering with help of the MNF
5	De-normalization
6	Gaussian filtering

First the Fourier-filtering is done, afterwards the barrel distortion is corrected. Before the noise removal with MNF the images for every wavelength are normalized to better find the noise. After the noise removal, the data set is denormalized again. As last step, a Gaussian filter is applied to further reduce the noise.

**Figure 1:**
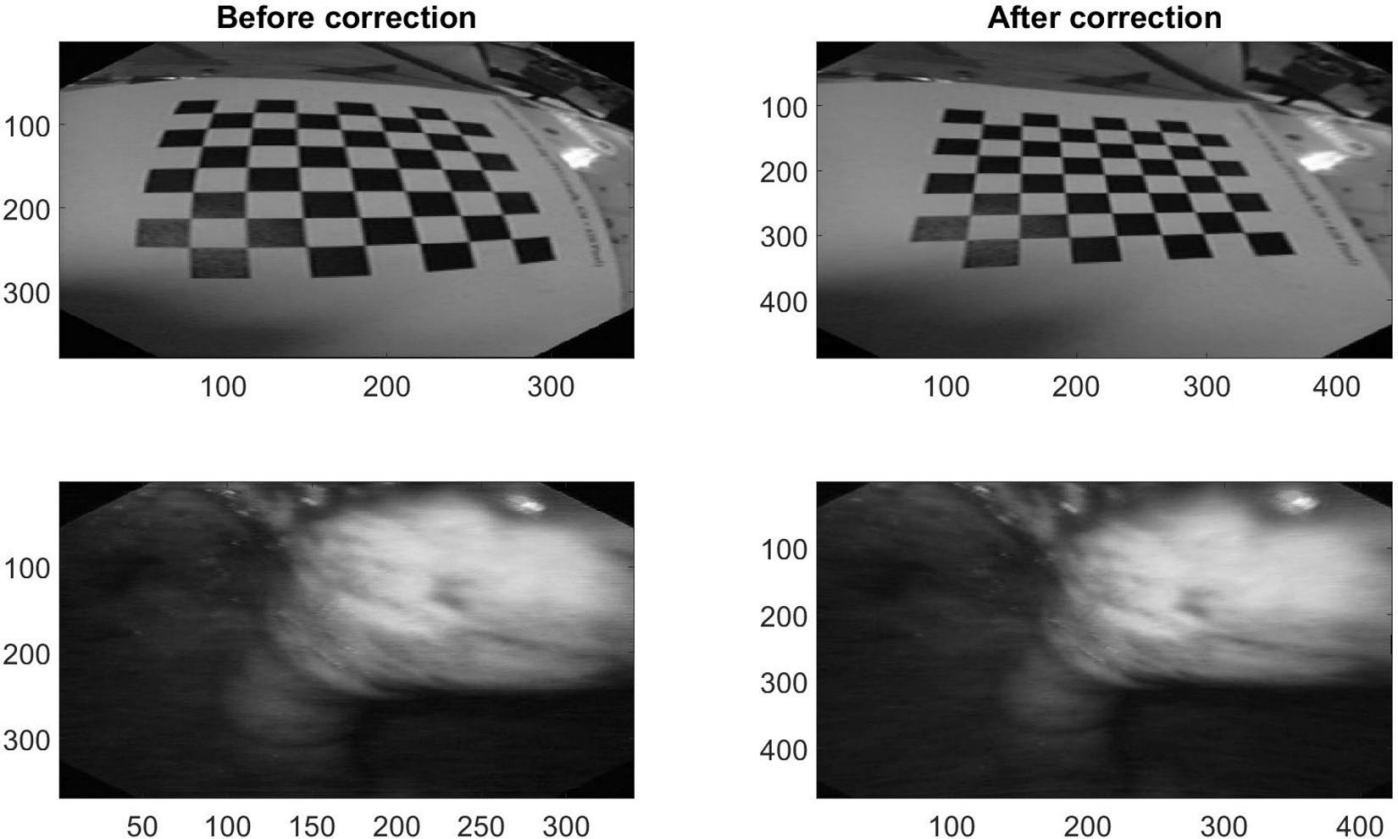
Example image of a checker board pattern and a carcinoma before and after correction of the barrel distortion.

The correction of the barrel distortion is done by imaging checker board patterns from eight different angles/distances with the endoscope and their detection is done by detecting the edge points of the checker board with the command “detectCheckerboardPoints” from Matlab. The detected points are used to calculate the world coordinate with the Matlab command “generateCheckerboardPoints”. As last point, the camera parameters are calculated by the command “estimateCameraParameters”. Figure 1 shows the original and the corrected image. The lines of the checker board are bent on the uncorrected image (left) and straight on the corrected image (right). This steps allows to use spatial features in the whole image.

### Spectral-spatial variation

The method of calculating the SSV is derived from the method of calculating the MNF transformation^12^. For the MNF, the most important point is the generation of the noise covariance matrix (NCM). In this study, the modified method from Regeling *et al*.^13^ is used. They^13^ adjusted a spectral and spatial decorrelation (SSDC) to the MNF which is according to Gao *et al*.^14^ the best method for NCM estimation for hyper spectral images.

For the estimation of the NCM, the image is divided into disjunct sub-images and a regressive SVM (rSVM) is used for deriving the noise as this method allows a better estimation of the noise than the method from Regeling *et al*.^13^. The rSVM is calculated by the “fitrlinear” command in Matlab. The size of the sub-images is varied from ten times ten to 100 times 100 pixels. For each sub-image, the rSVM is calculated and the difference between re-projection and the real data is regarded as the noise for NCM (equation 1):

1 $${r}_{i,j,k}={I}_{i,j,k}-{\hat{I}}_{i,j,k},$$

where *Î*_*i,j,k*_ is the re-projected value of *I*_*i,j,k*_ and the residuum (*r*_*i,j,k*_) is the difference between *I*_*i,j,k*_ and *Î*_*i,j,k*_. This difference is seen as the noise.

After the calculation of the residuum, I and r are reshaped into a matrix of the size of x-dimension of x times the y-dimension of x and the amount of wavelengths (x_size · y_size, lambda). The resulting matrices are named *R*_2_ and *X*_2_. First, the eigenvector expansion of the vectorized residuum matrix of the noise is calculated by singular value decomposition (SVD) which is shown in equation 2:

2 $${S}_{1}={U}_{1}\cdot {R}_{2}^{t}\cdot {R}_{2}\cdot {V}_{1}$$

where the columns of *V*_1_ are right-singular vectors and the columns of *U*_1_ are left-singular vectors.

The result from the SVD of the covariance matrix is used to whiten the original data:

3 $${W}_{X}={X}_{2}\cdot {U}_{1s}\cdot {({S}_{1}^{0.5})}^{-1}$$

where ^0.5^ is the element-wise square root and ^−1^ is the pseudo inverse of the matrix. The element-wise square root is used due to the fact that the singular values of the Matrix *R*_2_ (the non-zero elements of *s*_2_) are the square root of the positive eigenvalues of ${R}_{2}^{t}\cdot {R}_{2}$. From the whitened data the eigenvector expansion is calculated:

4 $${S}_{2}={U}_{2}\cdot {W}_{X}^{t}\cdot {W}_{X}\cdot {V}_{2}$$

This can be used now to derive the transformation matrix:

5 $${\rm{\Phi }}={U}_{1}\cdot {({S}_{1}^{0.5})}^{-1}\cdot {V}_{2}$$

As last step, the transformation matrix (Φ) has to be applied to the vectorized hyper spectral data-cube:

6 $$B={X}_{2}\cdot {\rm{\Phi }}$$

The resulting Matrix B are the components of the hyper spectral data-cube sorted by their signal-to-noise-ratio (SNR). Therefore, the higher order components can be set to zero and the noise reduced hyper spectral image can be derived by the following equation:

7 $$X={B}^{\ast }\cdot {{\rm{\Phi }}}^{-1}$$

where B^*^ is the matrix B where only the first 4, 5, 7 or 35 components are used. These values are chosen for covering a wider parameter range. This parameter is called MNF cut.

### Spatial features

For comparison to the SSV, normal spatial features are used. In this study, spatial features generated based on Laguerre-Gaussian functions are used. Typical spatial features are Gabor features which can be used as Morlet wavelets as shown from Bernadino *et al*.^15^. An example of Gabor features is the successful detection of cars by Arróspide *et al*.^16^. The Gabor features are well suited for the cars with their strong edges. However for carcinomas, the blood vessel pattern as the most important structural information for high magnifications and the gastric pits (GP) for normal endoscopy do not show this behaviour. To describe these patterns, better suited features should be chosen. To our knowledge, Laguerre-Gaussian functions allow this description quite well with a low amount of features.

Therefore after the pre-processing steps, Laguerre-Gaussian functions (LG) are used to create features. LGs are the multiplication of a Gaussian function with the Laguerre polynomials. The Laguerre polynomials are defined by the following formula:

8 $${L}_{p}^{l}(x)=\frac{{{\rm{e}}}^{x}\,{x}^{-l}}{p!}\,\frac{{d}^{p}}{d{x}^{p}}\,({{\rm{e}}}^{-x}\,{x}^{p+l})$$

where p is the p-th generalized Laguerre polynomial starting with p = 0. In this study, the Laguerre polynomials up to the order of l = p = 3 are used, leading to the following polynomials:

9 $${L}_{0}^{l}(x)=1$$

10 $${L}_{1}^{l}(x)=-\,x+l+1$$

11 $${L}_{2}^{l}(x)=\frac{1}{2}\,[{x}^{2}-2\,(l+2)\,x+(l+1)(l+2)]$$

12 $${L}_{3}^{l}(x)=\frac{1}{6}\,[-\,{x}^{3}+3\,(l+3)\,{x}^{2}-3\,(l+2)\,(l+3)\,x+(l+1)\,(l+2)\,(l+3)]$$

**Figure 2:**
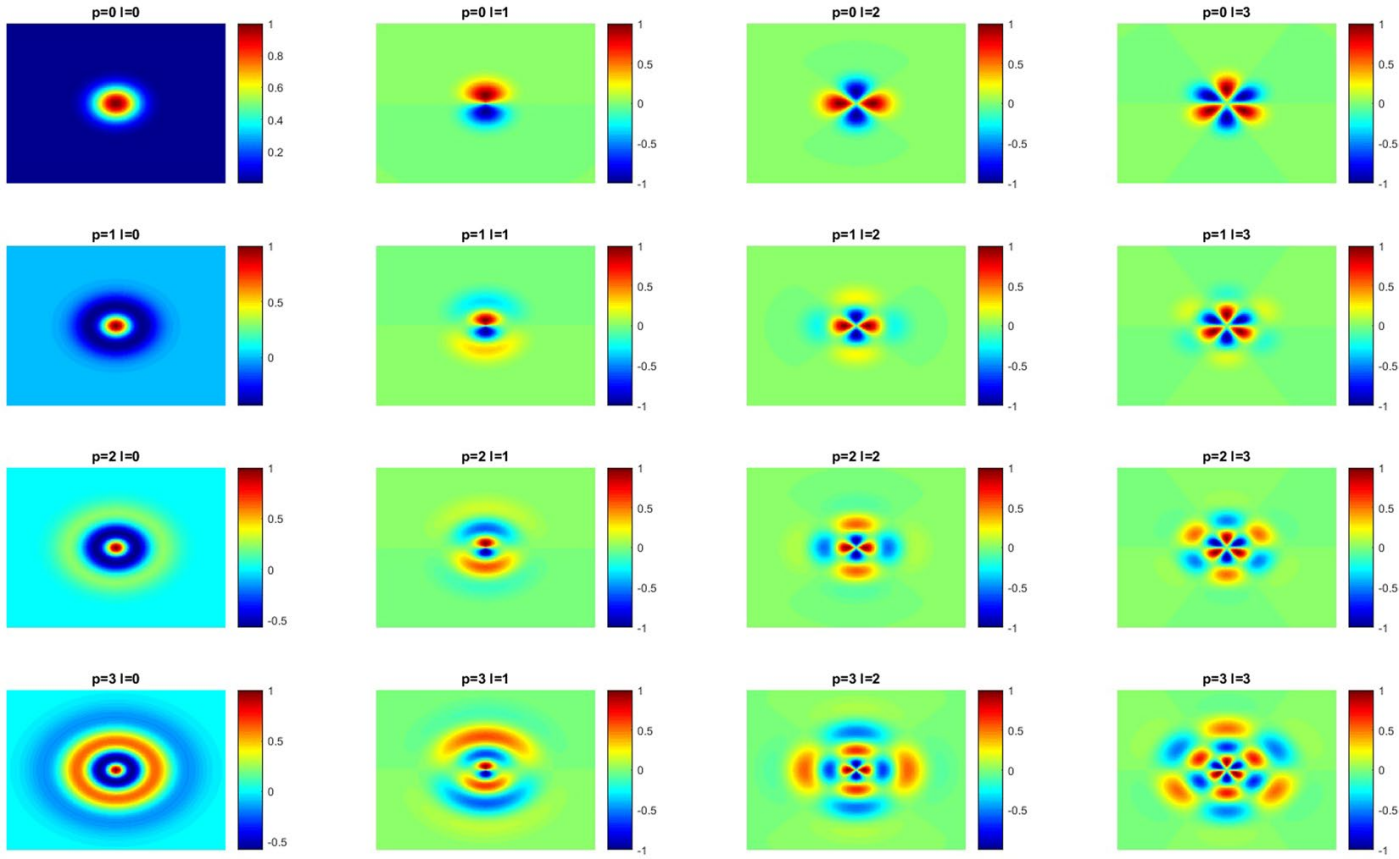
Laguerre Gaussian functions with varying l and p. On the upper left p and l are zero. Towards the right l is increased and towards the bottom p is increased. It should be noted that for *l* ≥ 1 the outer change is very small and therefore barely visible.

The polynomials are limited to p = l = 3 to reduce the calculation time and more importantly to reduce the memory usage. An example for the first LGs is shown in Fig. 2.

The LGs are generated with a resolution of 40 times 40 pixels and they are rotated in five steps for $\frac{180}{l}$ degree. The size of forty pixels is chosen as it is a compromise from not using to much of the surrounding and having enough pixels to represent the features well enough. Afterwards for every rotation, the convolution is calculated for each wavelength of the multi-spectral image. For each wavelength, the maximum of the absolute values for the different rotations is used as feature. This step is done to reduce the dimensionality of the data and to take into account that the same carcinoma might be imaged from different directions and/or distances. For the analysis, all features with *l*, *p* ≤ 3 are used. More features would lead to a too big amount of memory usage. In this study, the LGs as spatial features are leading to additional 72 features.

### Data analysis

For the ground truth, every carcinoma is confirmed by a biopsy. On each carcinoma, four to six biopsies are performed. The margin of the carcinoma is in all hyper spectral images marked manually by an medical expert. This leads to errors of the carcinoma margin which cannot be prevented currently. However, at the moment this is the only way to access the carcinoma margins in an endoscopic *in-vivo* setting. Therefore, the chosen classifiers should be robust against mislabelling. The same classifiers as in our previous study are used. These are AdaBoost (AB), SVM with linear and Gaussian kernel, random forest walk (RFW) and RobustBoost (RB). SVM is chosen due to the fact that it provides in general good results for hyper and multi spectral classification of carcinomas^17–19^. AB is used with a tree leaner because in many cases it provides good results. RB is used due to the fact that is compensates mislabelling^20^ as also shown in our previous study^5^. RFW is used due to the fact that it is robust against noise^21^ and shows sometimes better results than SVM for cancer classification^22^ or the analysis of hyper spectral images^23^.

The data analysis is done with the leave one out analysis. Therefore, n - 1 patients are used for training and the left out one for testing. This is done for all combinations. The training data is first used to do a principle component analysis (PCA). This is done of the complete data set. The PCA is done to reduce noise to improve the classification results, reduce the amount of features to speed up the classification and generate more reliable features in improve the classification results. Afterwards, 1% of the data is selected randomly for training of the classifiers to speed up the classification process. From each patient, 2–17 images are available. If one per cent of the data is used for training, this leads to about 75000 to 86000 data points for training.

All classifiers are evaluated with two different measures: the Matthews correlation coefficient (MCC^24^) is calculated:

13 $$MCC=\frac{TP\cdot TN-FP\cdot FN}{\sqrt{(TP+FP)(TP+FN)(TN+FP)(TN+FN)}},$$

where TN is true negative, TP is true positive, FN is false negative and FP is false positive. The MCC is a balanced measure of an accuracy-like metric. Its main advantage is that it can be used also for problems when the classes are of very different sizes. All results are optimized for having a high MCC due to the fact that it is stated to be the best available single value to characterize the classification in a single number according to Powers^25^. Additionally, the area under the curve (AUC) is used as an operation point free measure.

As last step, it is tested if the improvement is statistically significant. The comparison is done between all parameters. The statistical significance is tested with a five way ANOVA test for repeated measurements with the dimensions: classifiers, effect of the subimage size, effect of the filtering by the MNF, usage of spatial features and usage of the SSV. The ANOVA test is used due to the fact that it allows to use all five dimensions at the same time to provide a more significant result. However due to the extended calculation time, not all parameters are varied to the full extend.

## Results and Discussion

**Figure 3:**
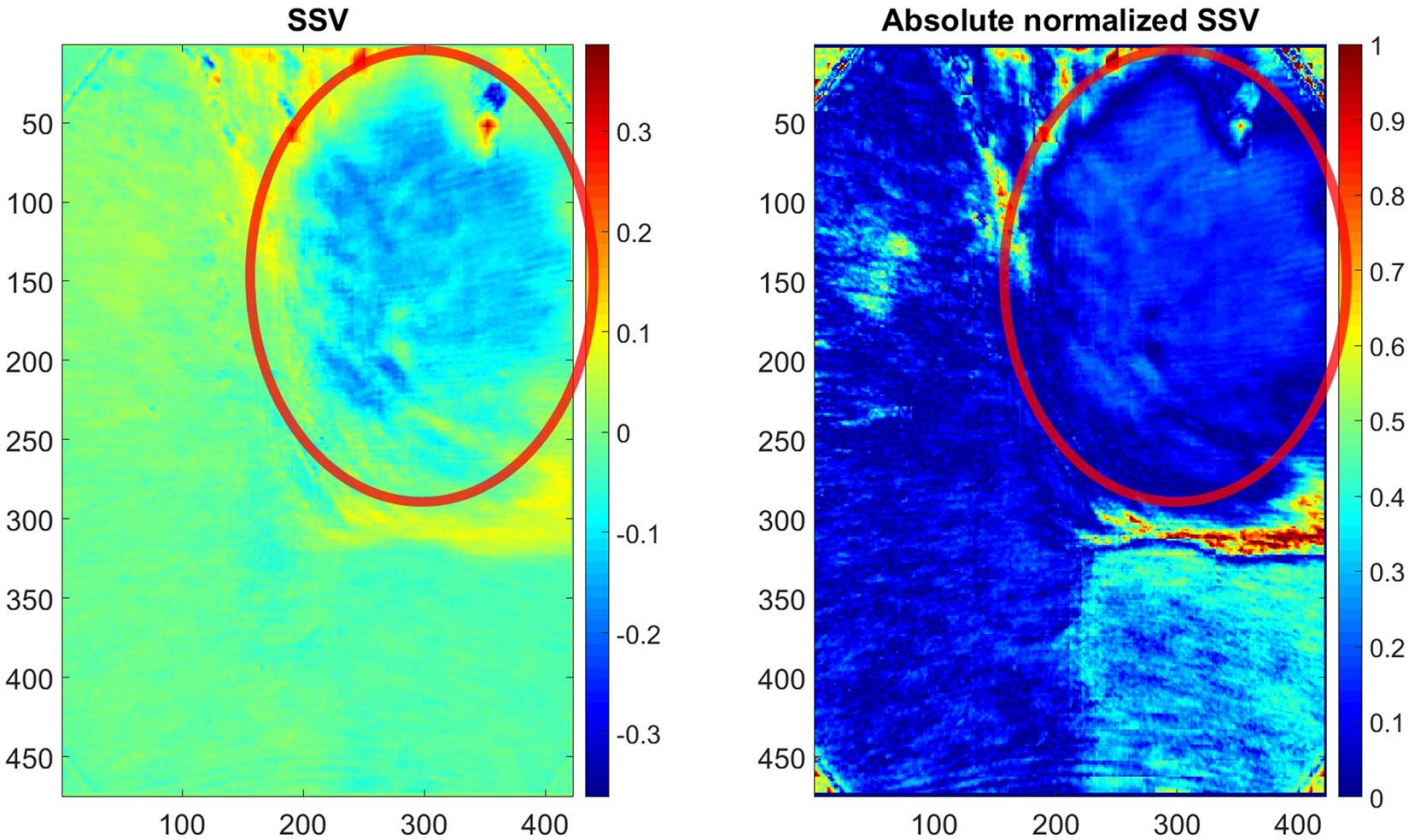
Example SSV (left) and absolute SSV normalized (divided) by the intensity of the original image (right). The red ellipses show the main area where the carcinoma is found.

First, the effect of the SSV is shown as an example image. Figure 3 shows on the left side the SSV and on the right side the absolute SSV normalized by the intensity of the original image. The red ellipse shows the main area of the carcinoma. The SSV is for most parts of the image constant except the upper right part. By dividing the SSV by the intensity of the image, the constant healthy tissue shows two different parts (Fig. 3 (right)). Thus, the SSV is at least partly independent of the intensity of the image. Thus for the healthy part, the SSV does not seem to depend on the intensity of the image. The upper right part is the area were the carcinoma is present and its SSV is clearly different (Fig. 3 (left)). The SSV is between around −0.03 and 0.045 in the healthy area at the areas without spectral reflection. The SSV of the cancerous area is around −0.18 to 0.02. Hence, there is a difference between them. However, it should be noted that most of the central carcinoma is in the range between −0.18 and −0.13.

The quite constant value of the SSV for the most parts of the image can be explained by the noise of the endoscope system and the normal spectral variance of the tissue. On the one hand, the endoscope system generates a constant noise of 5 intensity values for the whole image, therefore the SSV detects this variation. On the other hand, the carcinoma should have a different spatial and spectral variance than the healthy tissue. The different SSV for the carcinoma can be explained with three main points. First, this part of the image is closer to the camera of the endoscope and therefore more GPs are visible, leading in combination with movement artefacts to some higher SSV. Second, the movement artefacts are most likely caused by a stronger variance around the cancerous region when the movement artefacts alter the foreground with the background. Third and most important, the cancerous area itself might be the reason. At cancerous areas normally higher spatial and spectral variations are expected^26^. Thus, it indicates the presence of the cancerous tissue.

**Figure 4:**
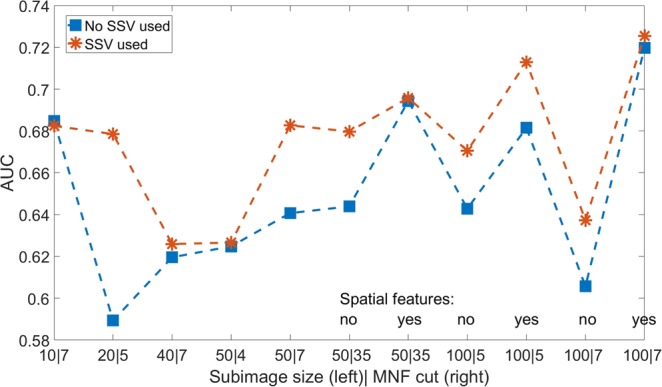
AUC as a function of the tested parameters: subimage size, MNF cut, spatial features and the usage of the SSV.

Figure 4 shows the mean result of the best classifier for comparison of the AUC of the parameter study for all parameters. There is an intersection of the two lines, representing the data with and without SSV, at only one specific data point (subimage size = 10 and MNF cut = 7). Thus, this main effect of the ANOVA should not be interpreted for SSV when this data point is included. However, it is only one point and a slightly overlap and it is for the mean result of the best classifier. Moreover, the statistical significance has to be analysed.

The analysis of a complete dataset for the AUC is shown in Table 2. The single parameters except the classifier show a significant effect. The classifier might not be significant as for the usage of strong filtering and spatial and spectral-spatial features, the boundaries might smear out and therefore RB cannot shine. Furthermore, there is an interaction between the subimage size, the MNF cut and the spatial features. The interaction between the MNF cut and the subimage size is expected as both parameters are generated from the calculation of the MNF. Moreover, both are parameters which alter the noise filtering of the MNF. The interaction of both of them with the spatial features is expected to have the same origin, as the generation of the spatial features adds 72 features which are a weighted average and therefore also some kind of noise reduction. However, the usage of the SSV does not show any interaction and seems to improve nearly all results. Hence, the SSV seems to be statistically independent of spatial features and the noise reduction. Therefore, there is significantly different information collected from the SSV compared to the standard spatial features.

**Table 2:** Anova for the AUC with the interaction as function of all tested parameters.

Source	Sum Sq.	d.f.	Mean Sq.	F	p
SSV	0.1211	1	0.12108	6.03	0.0142
MNF cut	0.2021	1	0.20213	10.1	0.0015
Subimage size	0.2028	1	0.20282	10.1	0.0015
Spatial features	0.1733	1	0.17334	8.64	0.0033
Classifier	0.0214	4	0.00535	0.27	0.8996
SSV*MNF cut	0.0466	1	0.04663	2.32	0.1277
SSV*Subimage size	0.0215	1	0.02152	1.07	0.3006
SSV*Spatial features	0.0094	1	0.00943	0.47	0.4933
SSV*Classifier	0.0137	4	0.00343	0.17	0.9533
MNF cut*Subimage size	0.1536	1	0.15358	7.65	0.0057
MNF cut*Spatial features	0.1778	1	0.17784	8.86	0.003
MNF cut*Classifier	0.0956	4	0.0239	1.19	0.313
Subimage size*Spatial features	0.1875	1	0.18747	9.34	0.0023
Subimage size*Classifier	0.0144	4	0.0036	0.18	0.9492
Spatial features*Classifier	0.0432	4	0.01079	0.54	0.7082

The SSV shows a significant effect on the AUC. The MNF cut, the subimage size and the usage of spatial features show also a significant effect and a significant interaction.

**Figure 5:**
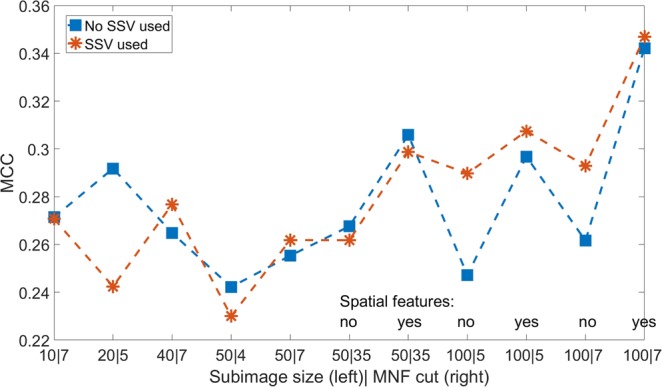
MCC as a function of the tested parameters: subimage size, MNF cut, spatial features and the usage of the SSV.

Figure 5 shows the mean result of the best classifier for comparison of the MCC of the parameter study for all parameters. The big amount of intersection already shows that an ANOVA cannot be used for the main effect of the SSV. Thus, no detailed analysis is shown.

## Conclusion

The usage of the SSV, as a new feature seems to be a proper way for classification of carcinomas. The method seems to work despite the quite high noise generated by the endoscope used in this study for all parameter combinations. Moreover, the information provided by the SSV uses the strong variance of the carcinomas to the advantage for detecting them instead of a hindrance. Also no interaction with other features is found. Therefore, the SSV might be used always as an additional feature set, too.

## References

[1] Crew K, Neugut A (2006). Epidemiology of gastric cancer. World Journal of Gastroenterology.

[2] Pohl J, May A, Rabenstein T, Pech O, Ell C (2007). Computed virtual chromoendoscopy: a new tool for enhancing tissue surface structures. Endoscopy.

[3] Qumseya B (2013). Advanced imaging technologies increase detection of dysplasia and neoplasia in patients with barrett’s esophagus: a meta-analysis and systematic review. Clinical Gastroenterology and Hepatology.

[4] Swager A, Curvers W, Bergman J (2015). Diagnosis by endoscopy and advanced imaging. Best Practice & Research Clinical Gastroenterology.

[5] Hohmann, M. *et al*. *In-vivo* multispectral video endoscopy towards *in-vivo* hyperspectral video endoscopy. *Journal of Biophotonics* (2016). 10.1002/jbio.201600021 27403639

[6] Yoshinaga S (2015). Evaluation of the margins of differentiated early gastric cancer by using conventional endoscopy. World journal of gastrointestinal endoscopy.

[7] Liedlgruber M, Uhl A (2011). Computer-aided decision support systems for endoscopy in the gastrointestinal tract: a review. IEEE reviews in biomedical engineering.

[8] Liu D-Y (2016). Identification of lesion images from gastrointestinal endoscope based on feature extraction of combinational methods with and without learning process. Medical image analysis.

[9] Zhang Q, Tian Y, Yang Y, Pan C (2015). Automatic spatial–spectral feature selection for hyperspectral image via discriminative sparse multimodal learning. IEEE Transactions on Geoscience and Remote Sensing.

[10] Fauvel M, Chanussot J, Benediktsson JA (2012). A spatial–spectral kernel-based approach for the classification of remote-sensing images. Pattern Recognition.

[11] Zhang L, Zhang L, Tao D, Huang X (2013). Tensor discriminative locality alignment for hyperspectral image spectral–spatial feature extraction. IEEE Transactions on Geoscience and Remote Sensing.

[12] Green AA, Berman M, Switzer P, Craig MD (1988). A transformation for ordering multispectral data in terms of image quality with implications for noise removal. IEEE Transactions on geoscience and remote sensing.

[13] Regeling, B. *et al*. Development of an image pre-processor for operational hyperspectral laryngeal cancer detection. *Journal of biophotonics* (2015). 10.1002/jbio.201500151 26033881

[14] Gao L, Du Q, Zhang B, Yang W, Wu Y (2013). A comparative study on linear regression-based noise estimation for hyperspectral imagery. IEEE Journal of Selected Topics in Applied Earth Observations and Remote Sensing.

[15] Bernardino, A. & Santos-Victor, J. A real-time gabor primal sketch for visual attention. In *Iberian Conference on Pattern Recognition and Image Analysis*, 335–342 (Springer, 2005).

[16] Arróspide, J. & Salgado, L. A study of feature combination for vehicle detection based on image processing. *The Scientific World Journal* **2014** (2014). 10.1155/2014/196251 PMC3932223 24672299

[17] Akbari H, Uto K, Kosugi Y, Kojima K, Tanaka N (2011). Cancer detection using infrared hyperspectral imaging. Cancer science.

[18] Akbari H (2012). Hyperspectral imaging and quantitative analysis for prostate cancer detection. Journal of biomedical optics.

[19] Akbari, H. *et al*. Detection of cancer metastasis using a novel macroscopic hyperspectral method. In *Medical Imaging 2012: Biomedical Applications in Molecular, Structural, and Functional Imaging*, vol. 8317, 831711 (International Society for Optics and Photonics, 2012). 10.1117/12.912026 PMC3546351 23336061

[20] Kobetski, M. & Sullivan, J. Improved boosting performance by explicit handling of ambiguous positive examples. In *Pattern Recognition Applications and Methods*, 17–37 (Springer, 2015).

[21] Breiman L (2001). Random forests. Machine learning.

[22] Statnikov A, Wang L, Aliferis CF (2008). A comprehensive comparison of random forests and support vector machines for microarray-based cancer classification. BMC bioinformatics.

[23] Pal M (2005). Random forest classifier for remote sensing classification. International Journal of Remote Sensing.

[24] Matthews BW (1975). Comparison of the predicted and observed secondary structure of t4 phage lysozyme. Biochimica et Biophysica Acta (BBA)-Protein Structure.

[25] Powers, D. Evaluation: From precision, recall and f-factor to roc, informedness, markedness & correlation’(spie-07-001). Tech. Rep., School of Informatics and Engineering, Flinders University, Adelaide, Australia (2007).

[26] Kiyotoki S (2013). New method for detection of gastric cancer by hyperspectral imaging: a pilot study. Journal of biomedical optics.

